# Molecular coupling competing with defects within insulator of the magnetic tunnel junction-based molecular spintronics devices

**DOI:** 10.1038/s41598-021-96477-3

**Published:** 2021-08-24

**Authors:** Pawan Tyagi, Hayden Brown, Andrew Grizzle, Christopher D’Angelo, Bishnu R. Dahal

**Affiliations:** Center for Nanotechnology Research and Education, Mechanical Engineering, University of the District of Columbia, Washington, DC 20008 USA

**Keywords:** Materials science, Nanoscience and technology, Physics

## Abstract

Nearly 70 years old dream of incorporating molecule as the device element is still challenged by competing defects in almost every experimentally tested molecular device approach. This paper focuses on the magnetic tunnel junction (MTJ) based molecular spintronics device (MTJMSD) method. An MTJMSD utilizes a tunnel barrier to ensure a robust and mass-producible physical gap between two ferromagnetic electrodes. MTJMSD approach may benefit from MTJ's industrial practices; however, the MTJMSD approach still needs to overcome additional challenges arising from the inclusion of magnetic molecules in conjunction with competing defects. Molecular device channels are covalently bonded between two ferromagnets across the insulating barrier. An insulating barrier may possess a variety of potential defects arising during the fabrication or operational phase. This paper describes an experimental and theoretical study of molecular coupling between ferromagnets in the presence of the competing coupling via an insulating tunnel barrier. We discuss the experimental observations of hillocks and pinhole-type defects producing inter-layer coupling that compete with molecular device elements. We performed theoretical simulations to encompass a wide range of competition between molecules and defects. Monte Carlo Simulation (MCS) was used for investigating the defect-induced inter-layer coupling on MTJMSD. Our research may help understand and design molecular spintronics devices utilizing various insulating spacers such as aluminum oxide (AlOx) and magnesium oxide (MgO) on a wide range of metal electrodes. This paper intends to provide practical insights for researchers intending to investigate the molecular device properties via the MTJMSD approach and do not have a background in magnetic tunnel junction fabrication.

## Introduction

Quest for utilizing molecule as a computer device element begun along with the mainstream silicon devices around seven decades before^1^. From 1950 to 1990, silicon technology grew exponentially while the lack of tools and technology forced molecular device research in its nascent state. From the early 1990, as attempts for making molecular devices gained momentum, the spintronics research grew tremendously and became commercially successful^2^. The parallel growth of two fields led to a new field of molecular spintronics^3^. Molecular spintronics devices (MSD) are promising for giving performance better than conventional silicon and spintronics devices^4^ and a candidate for providing hardware for quantum computers^5,6^. MSD based spin valves utilizing the electron's spin can be formed by chemically stitching molecular channels to the source and drains made up of magnetic materials or ferromagnets (FMs)^7,8^. However, it is a daunting task to maintain the molecule dimension (~ 0.5–3 nm) robust and reproducible gap between the two magnetic thin film layers^9–11^. Conventional molecular device fabrication approaches largely went backstage as they were not able to yield robust and mass-producible MSD based computer devices^3,9,11^. Specifically, the primitive MSD fabrication approach required placing the molecular monolayer between the electrodes^3^. However, molecular monolayer(s) are not dense and can be punctured due to inter-electrode metal diffusion and structural defects within molecules themselves^11^. Resultantly, defects within molecular monolayers create a direct exchange coupling that can compete or overturn the coupling via molecules and eventually may create a short circuit^3^.

Notably, most of the experimental molecular spintronics research has focused on nanogap devices. Such molecular devices are based on creating a break-junction in a nanowire to produce molecule length scale gap by electromigration^7,12^. However, the atoms that migrated during electromigration to create a nanogap may persist in the molecule's vicinity and create a competing coupling between the electrodes^13^. Additionally, mass production is extremely challenging due to the irreproducible break-junction profiles generated after electromigration or mechanical breaking of a nanowire.

**Figure 1:**
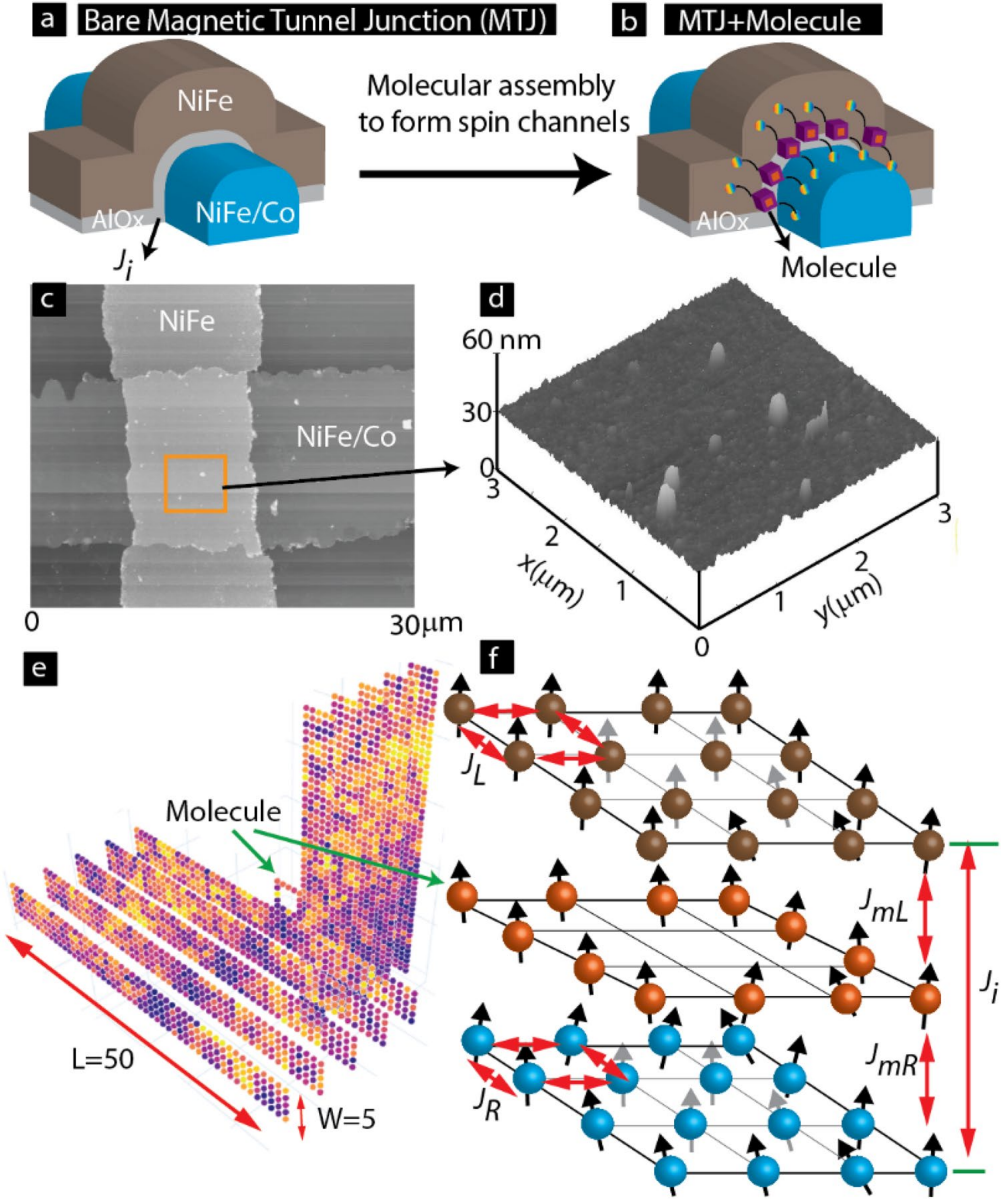
3D sketch of MTJ (**a**) before and (**b**) after connecting molecules between ferromagnets. AFM image of (**c**) an MTJ testbed with (**d**) hillocks like defects within junction area. (**e**) 3D Heisenberg atomic model of molecular device analogous to MTJMSD shown in panel (**b**). (**f**) Description of different coupling energy around the molecular junction and within ferromagnets of 3D model shown in panel (**e**). Sketches in panel (**a**), (**b**) and (**f**) are prepared in Adobe Illustrator CS6. Images (**c**) and (**d**) are obtained from Atomic Force Microscope. Sketch of 3D cartoon in (**e**) was produced from the indigenously written Python code https://colab.research.google.com/drive/1ACob9eSsKm3oH4y_X43rjLtVnfockUTv?usp=sharing. All the components were assembled in Adobe Illustrator CS6 version to produce the composite Figure.

To harness the industrial success of tunneling magnetoresistance devices^2^, we have explored a magnetic tunnel junction (MTJ) based MSD (i.e., MTJMSD approach). This MTJMSD method is intended to overcome limitations associated with conventional approaches. The MTJMSD is an excellent testbed for understanding and controlling the impact of coupling between the electrodes via defects in a molecular device^14^. This approach, utilizing commercially successful MTJ technology^2^ (Fig. 1a), is suitable for mass production and provides solutions to fabrication difficulties related to reliably connecting molecular device elements to the FMs^15^. The advanced design of MTJMSDs opens new possibilities, as described in a recent patent from our group^10^. For MTJMSD fabrication from a prefabricated MTJ, molecular channels are bridged across the insulator of an MTJ along the exposed side edges (Fig. 1b).

In an MTJMSD, by virtue of chemistry, the molecular channels are composed of covalently bonded atoms. Unlike conventional tunnel barrier materials possessing numerous pinholes and crystal-level defects, molecular channels are virtually defect-free. Interfacial defects between molecular channels and metal electrodes are expected to be negligent; we use strong Ni-thiol like chemical bonding to connect molecules to metal electrodes. Additionally, paramagnetic molecules reduce the effective tunneling barrier thickness to the length of the bridge between the metal electrode and molecule center ^15^. As a result, a highly coherent spin tunneling occurs via the molecular channel, and the physical tunneling barrier in the planner area becomes dormant^8^. As long as a robust insulating layer can be produced between two electrodes, MTJMSD can utilize any combinations out of semiconductor, ferromagnet, and non-metal. This flexibility can revolutionize the molecular device field because molecular device research is stuck mainly around gold^16^ and nickel^7,17^ over > 20 years. MTJMSD's top and the bottom electrodes can be deposited as a multilayer to alter the attributes of a metal layer with the help of an adjacent layer (Fig. 1a). For example, NiFe ferromagnetic films behave entirely differently when deposited on Cobalt (Co). A slight change in multilayer structure produced dramatically different properties with a paramagnetic molecule^18,19^. Additionally, MTJMSD can employ a wide range of tunneling barriers because molecular channels dominate transport and magnetic properties, and insulating barriers serve merely as a physical spacer.

In an MTJMSD, the insulator's quality and thickness play a dominant role in realizing the full potential of a molecule as the device element^20^. As a first condition, tunneling barrier thickness should be < molecule length. Under this condition, MTJMSD's ultrathin insulator should be able to yield minimum tunneling and remain stable for the device's lifetime. MTJMSD stability depends on various forms of mechanical stresses. Mechanical stresses can be generated during the growth of insulator and thin-film depositions, joul heating during transport studies, and environmental factors. Importantly, MTJMSD can utilize a vast range of electrodes and insulating materials. Each combination of metal electrodes and insulator in an MTJ will have to be fabricated, post-processed, and utilized to minimize mechanical stresses. Hence, the nature of defects and their impact can differ drastically and must be studied on a case-to-case basis. For example, with time ~ 2 nm AlOx deposited on NiFe FM electrode was much more stable than that deposited on Co^21,22^. Other tunneling barriers, such as MgO^23^, may perform very well for one metal and may appear unsuitable or challenging on other ferromagnets^24^. Mechanical stress relaxation can lead to numerous interfacial defects^24^ and irregularities within the tunneling barrier that can later compete with molecular channels. There is a lack of knowledge about the role of defects and how to characterize and understand them in the context of molecular spintronics devices.

This paper extensively discusses the example of the AlOx tunnel barrier-based MTJMSDs and elaborates a diagnostic method to identify the evolution of defects using a ferromagnetic resonance study. In the case of AlOx, we observed that several hillock-like features appear over a period after the MTJ testbed fabrication (Fig. 1c). The height of hillocks varied significantly across the junction area (Fig. 1d). Hence, the net property of the MTJMSD was the cumulative effect of coupling via molecules and any form of defects present in an insulator. In prior work, theoretical^25^ and experimental^20,26^ studies were performed to investigate the effect of defects on tunnel junctions. However, there is a void of knowledge about the influence of defects on MTJMSDs. This study reports experimental observations of competition between molecular device elements and defects. We theoretically explored the effect of coupling via an insulator on MTJMSD properties using Monte Carlo Simulation (MCS) methods to create a general understanding.

## Methods

We have studied the Heisenberg model of MTJMSD (Fig. 1e) to systematically investigate the effect of defect-induced exchange coupling (*J*_*i*_) in the presence of molecule-induced exchange coupling with ferromagnets (Fig. 1f). This paper elaborates experimental and theoretical data in the context of competing coupling via defects and molecules for MTJMSD. The experimental fabrication process for MTJMSD has been discussed elsewhere^8,14,15,19,27^. Prior work provides experimental details of patterning, depositing ferromagnetic electrodes, insulating barrier, and molecular attachment method^8,14,15,19,27^. The simulation study in this paper is based on the design of experimentally researched MTJMSDs (Fig. 1b). In our experimental research, MTJMSD was formed by connecting organometallic molecular clusters (OMC)^28^ between ferromagnets^15,19^. Every part of MTJMSD, i.e., molecules, FM, and electrodes, is represented by the Heisenberg model (Fig. 1e,f).

The rationale for adopting parametric MCS simulation to understand the role defects on MTJMSD are the following. (i) Our prior MCS study provided insights about the intriguing experimental observations on MTJMSDs for the cases where paramagnetic molecules produced strong exchange coupling^18^. It is noteworthy that we could also explain dramatic changes in cross-junction-shaped MTJMSD transport using the MCS model^8^. (ii) We did not pursue DFT to explain and understand near room temperature behavior of MTJMSD. Conventionally used DFT approaches fundamentally work for absolute zero temperature. DFT approach is unable to simulate the effect of a wide range of thermal energy on an MTJMSD. Studying the role of thermal energy is critical because magnetic materials and magnetic molecules change dramatically with temperature^28–30^. (iii) Also, DFT is exceptionally challenging, if not impossible, to simulate the role of a wide range of defects in a system comprising thousands of transition metal atoms in a magnetic tunnel junction and molecular magnets. By varying the defect-associated parameter in MCS, *J*_*i*_ in Fig. 1f, we have attempted a wide range of possibilities without delving into a prohibitive list of potential variations of defects. (iv) The basis of our assumption of representing defects with *J*_*i*_ is consistent with the prior theoretical simulation on MTJ. In the prior work, defects within the tunnel barrier impacted the exchange coupling (akin to *J*_*i*_ in this paper) between two FM electrodes of an MTJ^25^. (v) We were unable to find a simulation method that could simulate the role of defects for the case of molecule-induced strong exchange coupling as experimentally observed in our prior work^18,19,31^. By parametrically varying molecular coupling factors (*J*_*mL*_ and *J*_*mR*_ Fig. 1f) we could simulate a wide range of possibilities from weak to strong exchange coupling. (vi) In the present MCS simulations, we have represented complex molecules with atomic analogs. Our assumption of simplifying complex molecules is based on the prior work utilizing conventional single electron physics to represent various molecules^32–34^. Molecules behaved like a single electron device element and followed the associated physics in experimental observations^34^.

To practically accomplish the MCS study with desktop workstations in a reasonable time, the size of the MTJMSD Heisenberg model was fixed within $11\times 50\times 50$ 3D atomic space (Fig. 1e). MTJMSD Heisenberg model has 11 atomic layers. The central layer corresponds to the molecular layer (Fig. 1e). In the MTJMSD model, FM electrodes are of $5\mathrm{ atom}\times 5\mathrm{ atom}\times 50$ atom, and molecules are represented by the $5\mathrm{ atom}\times 5\mathrm{ atom}$ square with an empty interior (Fig. 1e). To describe the molecules on the edges (Fig. 1b), a plane containing atoms with an empty interior was introduced between the two FM electrodes at the cross junction (Fig. 1f). A similar approach has been applied in our prior work producing simulation results for pillar-shaped MTJMSD (i.e., FM electrodes are of the same dimensions as of the junction area) conforming with experimental observations^18^. The inter-FM electrode magnetic coupling occurs via the molecules and via the insulating layer between two FM electrodes (Fig. 1f). However, inter-FM electrode coupling via the insulating layer is defined by the exchange coupling parameter *J*_*i*_. We performed MCS by varying molecular coupling strength with the left FM (*J*_*mL*_) and right FM (*J*_*mR*_) electrodes.

We varied thermal energy $(\mathrm{kT}$) to simulate the temperature-dependent property of MTJMSD. Assuming 500–1300 °C is the Curie temperature range for most of the ferromagnets, $\mathrm{kT}=0.1$ is equivalent to 50–130 °C temperature range. To achieve the MTJMSD equilibrium state during the MCS study, we minimized the system energy as defined in Eq. (1). Our MC studies utilized a continuous model^35^. The continuous model allowed spin vectors to settle in any randomly selected direction in a spherical coordinate system and hence have 3D vector components according to the equilibrium energy governed by Eq. (1).

1 $$E=-{J}_{L}\left({\sum }_{i\in L}{\overrightarrow{S}}_{i}{\overrightarrow{S}}_{i+1}\right)-{J}_{R}\left({\sum }_{i\in R}{\overrightarrow{S}}_{i}{\overrightarrow{S}}_{i+1}\right)-{J}_{mL}\left({\sum }_{i\in L,i+1\in mol}{\overrightarrow{S}}_{i}{\overrightarrow{S}}_{i+1}\right)-{J}_{mR}\left({\sum }_{i-1\in mol,i\in R}{\overrightarrow{S}}_{i-1}{\overrightarrow{S}}_{i}\right)-{J}_{i}\left({\sum }_{i\in L,i+1\in R}{\overrightarrow{S}}_{i}{\overrightarrow{S}}_{i+1}\right)$$

where *S* represents the spin of individual atoms of FM electrodes and molecules in the form of 3D vectors, *J*_*L*_, and *J*_*R*_, are the Heisenberg exchange coupling strengths for the FM electrodes on the left and right FM electrodes. For all MCS, the boundary condition was selected so that the atomic spin beyond the boundary atom of the MTJMSD model (Fig. 1e) was zero^35^. After choosing appropriate values for the Heisenberg exchange coupling coefficients, $kT$, and random spin states, a Markov process was set up to generate a new state. Detailed description of our MCS is published elsewhere^18^. Every MC simulation was run for 200–500 million steps to achieve a stable low energy state. After achieving the equilibrium states, additional iterations were performed to generate an average magnitude of observables based on several thousands of equilibrium data^35^. The units of total energy $E$ and exchange coupling parameters are the same as of $kT$. To keep the discussion generic, the exchange coupling parameters and $kT$ are referred to as the unitless parameters throughout this study. The overall magnetic moment of the MTJMSD is the sum of the magnetic moment of the two FM electrodes and the magnetic moment of the molecules.

## Results and discussions

**Figure 2:**
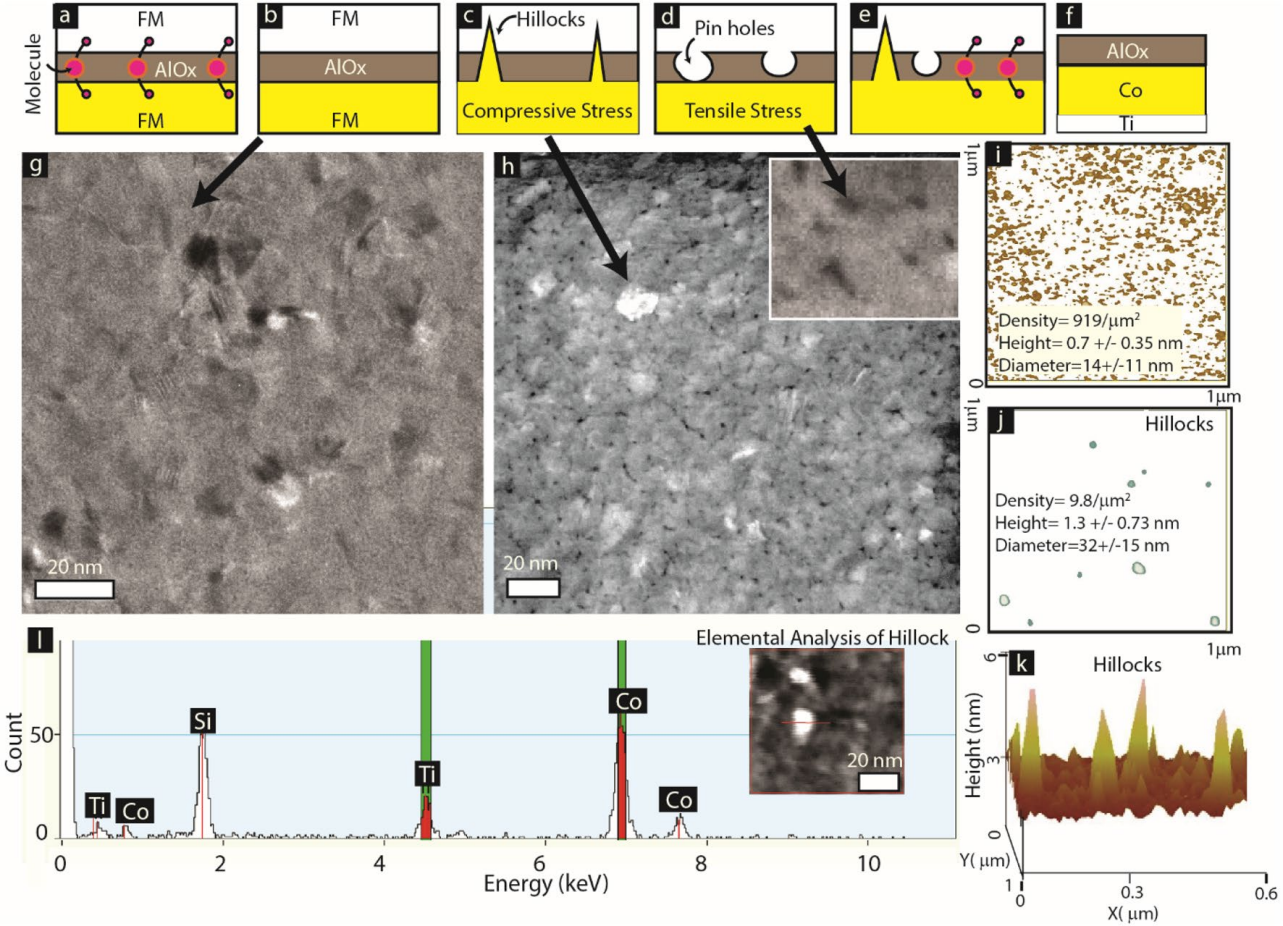
Conceptual sketches showing cross-sectional sideview of (**a**) an MTJMSD with defect free insulator, (**b**) MTJ testbed with defect free insulator, (**c**) MTJ with hillocks, (**d**) MTJ with pin holes, (**e**) an MTJMSD with competing molecule and defects. (**f**) Testbed used for investigating defects in AlOx. TEM image of (**g**) as produced and (**h**) thermally stressed Ti/Co/AlOx. Representative AFM image analysis quantify (**i**) pinholes, and (**j**) hillocks. (**l**) Compositional analysis confirming hillocks are made up of cobalt (Co) and Ti. Inset image in (**l**) correspond the site of compositional analysis in the TEM study. (**k**) 3D AFM image showing sharp hillocks. Panel (**a**–**e**) were sketched in Adobe Illustrator CS6. (**a**–**e**) Sketches, TEM images in panel (**g**, **h**), and and atomic force microscope images (**i**), (**j**), and (**k**) were assembled in Adobe Illustrator CS6.

The foundational concept of this paper is based on the experimentally observed defects in the tunnel barrier of an MTJMSD (Fig. 2). Data in Fig. 2a depicts an MTJMSD system where molecular channels can impart the maximum impact as an exchange coupling agent, which is possible because the MTJ used in this case includes a perfect tunnel barrier (Fig. 2b). However, this is hardly the case, and almost no tunnel junction is without some kind of structural defects. For instance, a family of metallic electrodes can produce compressive stress-induced hillocks. Such hillocks lead to localized short circuits or relatively high transport. These stress-induced hillocks become the basis of defect-induced exchange coupling (*J*_*i*_) competing with molecules. The growth mechanism of the ultra-thin insulator on various types of the substrate may lead to non-uniform thickness across the planar area. Also, interfacial mismatch strains may lead to point defects^24^ becoming a source of *J*_*i*_. Additionally, the residual tensile stress during fabrication, annealing^22^, and charge transport^26,36^ may cause the tunnel barrier fragmentation, producing pinholes-like features. Such defects can become the basis of direct exchange coupling between FM electrodes (*J*_*i*_) (Fig. 2d). Hence, it is likely that an MTJMSD may have various defects competing with molecular coupling (i.e., *J*_*mL*_ and *J*_*mR*_).

To provide insights about the role of representative defects in MTJMSD here, we provide microscopy images collected on a system of cobalt (Co, yellow layer) under ~ 2 nm AlOx insulator (Fig. 2f). The top electrode is avoided for direct observations of defects in the Ti(5 nm)/Co(10 nm)/AlOx(2 nm) stack; Ti served as adhesion between substrate and Co. The top view collected from Transmission Electron Microscopy (TEM) shows that even as produced, ultrathin AlOx contained some depressions and dark white spots (Fig. 1g). The depression areas are representing zones where the thickness of AlOx is lower than ~ 2 nm. The white color regions are attributed to the compressive stress-induced hillock generation^20^. It is critical to note that the density of defect scale with tunnel junction area. The same AlOx sample showed an increased number of defects after heating by the high-energy electron beam in TEM (Fig. 2h). In Fig. 2h, the AlOx surface had the ubiquitous presence of cracks-like features; the zoomed-in view of the crack resembles the pinholes-like feature shown in Fig. 2d. According to AFM imaging-based quantitative estimation, AlOx surface possessed ~ 919/µm^2^ pinholes. The pinholes were 0.7 ± 0.3 nm high with respect to Co/AlOx interface. The pinhole diameter was 14 ± 0.35 nm. Similarly, TEM observed several white regions (Fig. 2h) representing the compressive stress-induced hillocks (Fig. 2c). AFM analysis showed that these hillocks-like features were ~ 9.8/µm^2^. These hillocks-like features had 1.3 ± 0.7 nm height and 32 ± 15 nm diameter (Fig. 2k). The 3D view shows that hillocks could have sharp features (Fig. 2l). These features could poke through the insulating layer and made the connection with the top electrode in case of complete tunnel junction (Fig. 1d). The Energy dispersive x-ray spectroscopy (EDS) analysis also confirmed that hillocks-like features are made up of Co (Fig. 2l); Co present under the AlOx served as the compressible metal that could shoot out and puncturing the AlOx. By no means do we claim our quantitative analysis is reproducible or exact; however, the presence of some form of defects, hence the defect-induced direct coupling (*J*_*i*_) between the top and bottom electrodes, is inevitable.

We performed Ferromagnetic Resonance (FMR) to investigate the effect of the defects in an MTJ before and after establishing molecular channels along the edges. For this study, we mainly utilized samples with several thousand cylindrical-shaped ~ 4 µm diameter MTJs. A large MTJ population produced strong FMR signals enabling unambiguous FMR mode detection from the as-received data. There are innumerable possibilities in MTJ types; however, to get the gist of defect induced *J*_*i*_, we mainly studied MTJ with SiO_2_(substrate)/Ta(2 nm)/Co (5–7 nm)/NiFe(5 nm)/AlOx(~ 2 nm)/NiFe(10 nm) thin-film configuration. The shape of cylinders was defined photolithographically. We deposited all the metallic layers by a sputtering process using our optimum fabrication process reported elsewhere^18^.

**Figure 3:**
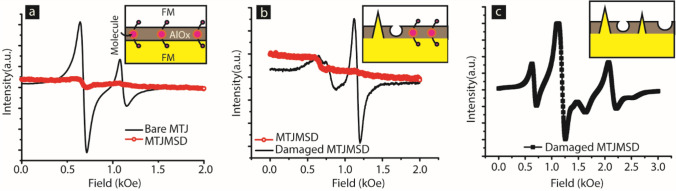
FMR experiment monitoring the position and intensity of modes on a (**a**) good MTJ sample transforming into stable MTJMSD, (**b**) a good MTJMSD failed under mechanical stress, and (**c**) an exemplary MTJ with damaged tunneling barrier. FMR data in panel (**a**), (**b**), and (**c**) were plotted in Originlab^®^ 2016 softawre. Inset sketches in each panel is drawn in Adobe Illustrator CS6^®^. All the components were assembled and labelled in Adobe Illustrator CS6^®^.

We referred to the prior literature for the FMR data analysis for coupled ferromagnets^37^. Coupled ferromagnets typically produced two modes: acoustic resonance mode (FMR mode with higher intensity) and optical resonance mode (FMR Resonance mode with lower intensity)^37^. MTJ with good quality tunnel barrier, presumably with structural properties similar to the micrograph (Fig. 2g), produced discernible FMR modes (Fig. 3a). The stable MTJ in the bare state produced optical mode (lower intensity peak) appearing before acoustic mode (high-intensity peak) (Fig. 3a). Such resonance mode profile corresponds to the weak antiferromagnetic coupling. These results provide direct evidence that presumably good quality ~ 2 nm AlOx produces a finite amount of exchange coupling, and hence certainly, *J*_*i*_ exists. It is noteworthy that in the present case, *J*_*i*_ represents the direct coupling between the two FM electrodes of the MTJ in the bare state; ~ 2 nm AlOx grown with optimum fabrication protocol is unable to create perfect isolation. Future development in MTJMSD needing *J*_*i*_ = 0 may have to reengineer MTJMSD and focus on utilizing different insulators and better insulator fabrication processes^10^.

Bare MTJ was treated with paramagnetic molecules to create molecular channels along the exposed side edges, like the conceptual sketch shown in Fig. 3a. The details of paramagnetic molecules are published elsewhere^29,30^. A radically different FMR signal was observed after transforming MTJ into MTJMSD by creating molecular channels along the exposed side edges produced (Fig. 3a). MTJMSD showed almost complete disappearance of the acoustic and optical modes at room temperature. The mode disappearance was calculated in the extreme cases of antiferromagnetic coupling between ferromagnetic electrodes^38,39^. This study suggests paramagnetic molecules produced extremely strong antiferromagnetic coupling on an MTJ testbed that showed weak antiferromagnetic coupling via AlOx (Fig. 3a).

Here we also report the effect of mechanical stress-induced defects^20^. The sample for the FMR study was produced when we mechanically bent the MTJMSD chip to break it into smaller pieces. A broken piece, part of a bigger sample showing FMR shown in Fig. 3a, started exhibiting a new FMR modes configuration (Fig. 3b). New FMR mode configuration corresponds to ferromagnetic coupling^37^. The appearance of optical mode before acoustic resonance mode on damaged MTJMSD signifies that *J*_*i*_ is ferromagnetic coupling type^40^. Hence, defect-induced ferromagnetic coupling negated the molecule-induced strong antiferromagnetic coupling. An in-depth discussion about the nature and quantity of defects is beyond the scope of this paper and may not be adding new insights. The more important lesson is that defects can produce a strong *J*_*i*_ that can nullify or negate the paramagnetic molecule-induced exchange coupling. The conceptual depiction of co-existing *J*_*i*_ and molecule induced *J*_*mL*_ and *J*_*mR*_ exchange coupling with the two electrodes is shown in Fig. 3b. Occasionally, we observed that samples produced with accidental deviations in optimum fabrication methods resulted in a large variety of FMR signals (Fig. 3c).

We have provided several intriguing observations resulting from molecule-induced strong antiferromagnetic coupling elsewhere^18,19,31,41^. The most striking observation is the room temperature current suppression phenomenon with cross junction shaped MTJMSD (Fig. 1a,b)^14^. In the context of the present paper, it is critical to visualize the impact of defect-induced coupling on MTJMSD charge transport. After a tunnel barrier failure, an MTJ stabilized in a high current state. The high current state on failed MTJs suggests that defects produced a ferromagnetic type coupling via the site of defects. Therefore, an MTJMSD that shows room temperature current suppression must have a tunnel barrier with defects not producing significant competition (i.e., ferromagnetic coupling). Defects producing net ferromagnetic coupling are not expected to show stable current suppression.

This paper has included and discussed the transport study to highlight the impact of defect-induced coupling on MTJMSD (Supplementary Fig. S1). Typically, a stable MTJ with ~ 25 µm^2^ junction area and ~ 2 nm AlOx produce less than 0.5 µA current at 100 mV bias. An MTJ showing 10–100 times higher leakage current under identical conditions signifies the dominant presence of defects within ~ 2 nm tunnel barrier (Supplementary Fig. S1a). High current on defective MTJ also corroborates with the conclusion that defects typically produced magnetic coupling with ferromagnetic nature (Fig. 3a,c). An MTJ with a defective tunnel barrier with a 3–10 µA level leakage current never produced a stable current after hosting paramagnetic molecules (Fig. 3b). Such MTJMSDs with defective tunneling barriers showed a random extent of current decrease compared to the bare MTJ (Supplementary Fig. S1b). It is presumably due to the competing defect-induced exchange coupling (*J*_*i*_) and paramagnetic molecule-induced exchange coupling with two ferromagnetic electrodes Fig. 1f. On the contrary, a stable MTJ with < 0.5 µA leakage current showed a significant increase in current after hosting molecular channels between two FM electrodes and with few minutes to several hours, settled in a suppressed current state (Supplementary Fig. S1c). The suppressed current state where paramagnetic molecule-induced exchange coupling was manifesting its full potential was 3–6 orders of magnitude lower than the bare MTJ leakage current level (Supplementary Fig. S1d). It is obvious that molecule coupling can show the effect with a low level of defect-induced exchange coupling. As shown in the TEM micrograph, even freshly produced AlOx tunnel barrier possessed some defects. There is no possibility of having a defect-free tunnel barrier (Fig. 2g).

To understand the impact of defect-induced exchange coupling on MTJMSD, we performed Monte Carlo simulations (MCS). MCS studies investigating the effect of a wide range of *J*_*i*_ and Molecular coupling (*J*_*mL*_ and *J*_*mR*_) provide the opportunity to understand a vast range of MTJMSD that can be produced by combining various types of MTJ and paramagnetic molecules with uncertain amount and nature of defects. We conducted MCS to investigate the effect of defect-induced *J*_*i*_, without delving into the nature of defects. We varied *J*_*i*_ for different cases of molecule-induced exchange coupling. The positive sign of *J*_*i*_ indicated ferromagnetic coupling, and the negative sign indicated antiferromagnetic type coupling.

**Figure 4:**
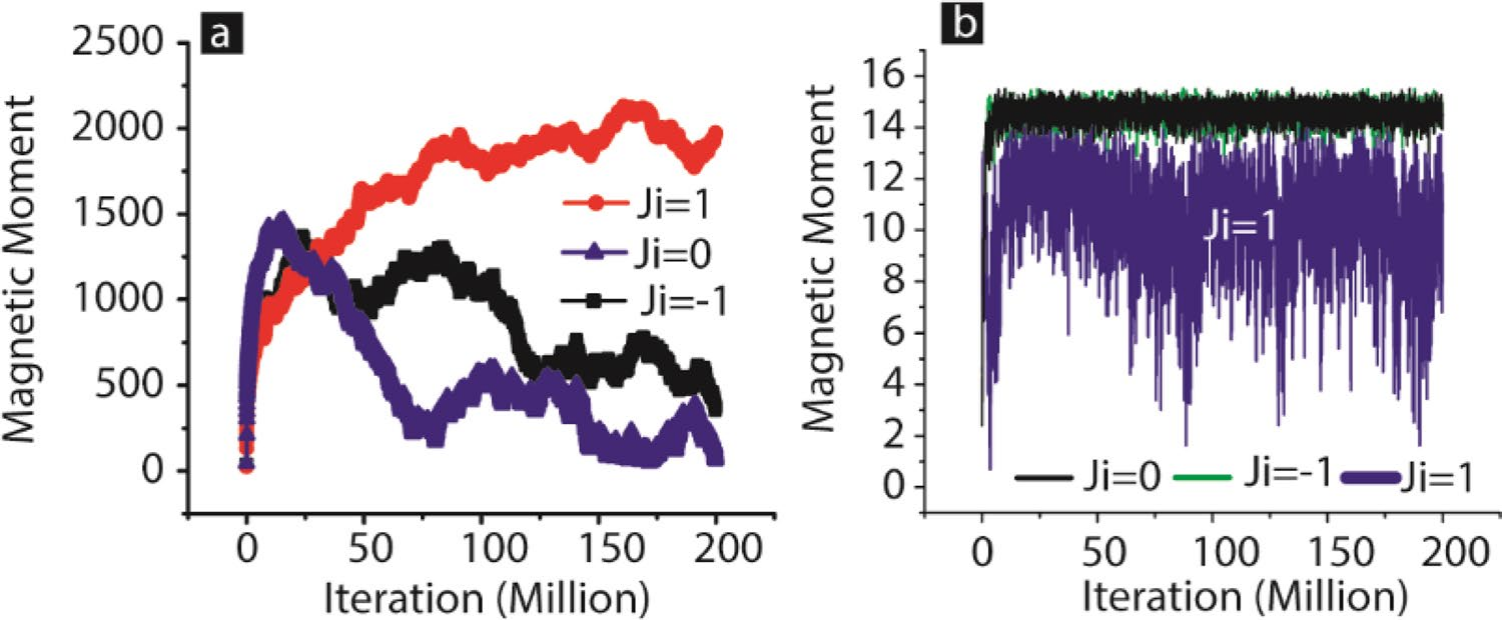
Temporal evolution of (**a**) MTJMSDs, and (**b**) molecules magnetic moment under *J*_*i*_ = 1, 0, and − 1. The simulations are performed for *kT* = 0.1 and molecule induced strong exchange coupling. Simulation data is plotted in Originlab^®^ 2016 and assembled in Adobe Illustrator^®^ CS6.

Initially, we studied the impact of *J*_*i*_ on the MTJMSD evolution process to equilibrium state; in this study, we maintained strong molecule-induced exchange coupling and *kT* = 0.1. To highlight the impact of *J*_*i*_ here, we mainly report MTJMSD, where molecule produced strong antiferromagnetic coupling. Molecule produced a net antiferromagnetic coupling when the sign of *J*_*mL*_ and *J*_*mR*_ was opposite, and magnitude was equal to 1. We recorded the magnetic moment of MTJMSD during the time span for which it evolved from random spin orientation to the equilibrium state (Fig. 3a). The MTJMSD's maximum magnetic moment can ~ 2500 when all the FM atoms and molecules are aligned in the same direction. On the other hand, MTJMSD's lowest magnetic moment is due to 16 molecules when two equal dimensions ferromagnetic electrodes are perfectly aligned opposite each other. For *J*_*i*_ = 0 cases, after 200 million iterations, MTJMSD settled into only a magnetic moment state with a magnitude of 500. The MTJMSD showing a magnetic movement of 500 is because at finite temperature, not every atom is aligned in the same direction in 3D space. For *J*_*i*_ = 1 case, the total magnetic moment of the device settles nearly close to 2000. It means defect-induced ferromagnetic exchange coupling has overcome the impact of molecule-induced strong antiferromagnetic coupling (Fig. 4a). This theoretical calculation provides insights about the experimental observation reported in Fig. 4. Experimentally we saw that defect produced a net ferromagnetic coupling that generally forced an MTJMSD to settle into a high current state.

We also simulated the case when defect could produce antiferromagnetic coupling between the ferromagnetic electrodes of the MTJMSD. For *J*_*i*_ = − 1, representing the strong antiferromagnetic coupling due to the defects, MTJMSD settled into the magnetic moment state with a magnitude of near 16 (Fig. 4a). These results suggest that defect-induced antiferromagnetic coupling complemented the molecule-induced antiferromagnetic coupling. Strong defect-induced antiferromagnetic coupling produced a stronger effect, enabling MTJMSD to settle into the lower magnetic moment state quicker (Fig. 4a). We also became curious about the molecule's magnetic moment during the equilibrium state settlement under different defect-induced exchange coupling scenarios. When *J*_*i*_ was negative, the magnetic moment of the molecules was ~ 16 (Fig. 4b).

Interestingly, for positive and strong *J*_*i*_, the molecules' magnetic movement was unsteady throughout the simulation and varied between 14 and 2 (Fig. 4b). Our MCS provides a mechanistic understanding of how defect-induced exchange coupling works when it is the opposite of the induced exchange coupling. *J*_*i*_ disturbed the coherence of molecules' magnetic moment and hence nullifying the effect of their presence.

**Figure 5:**
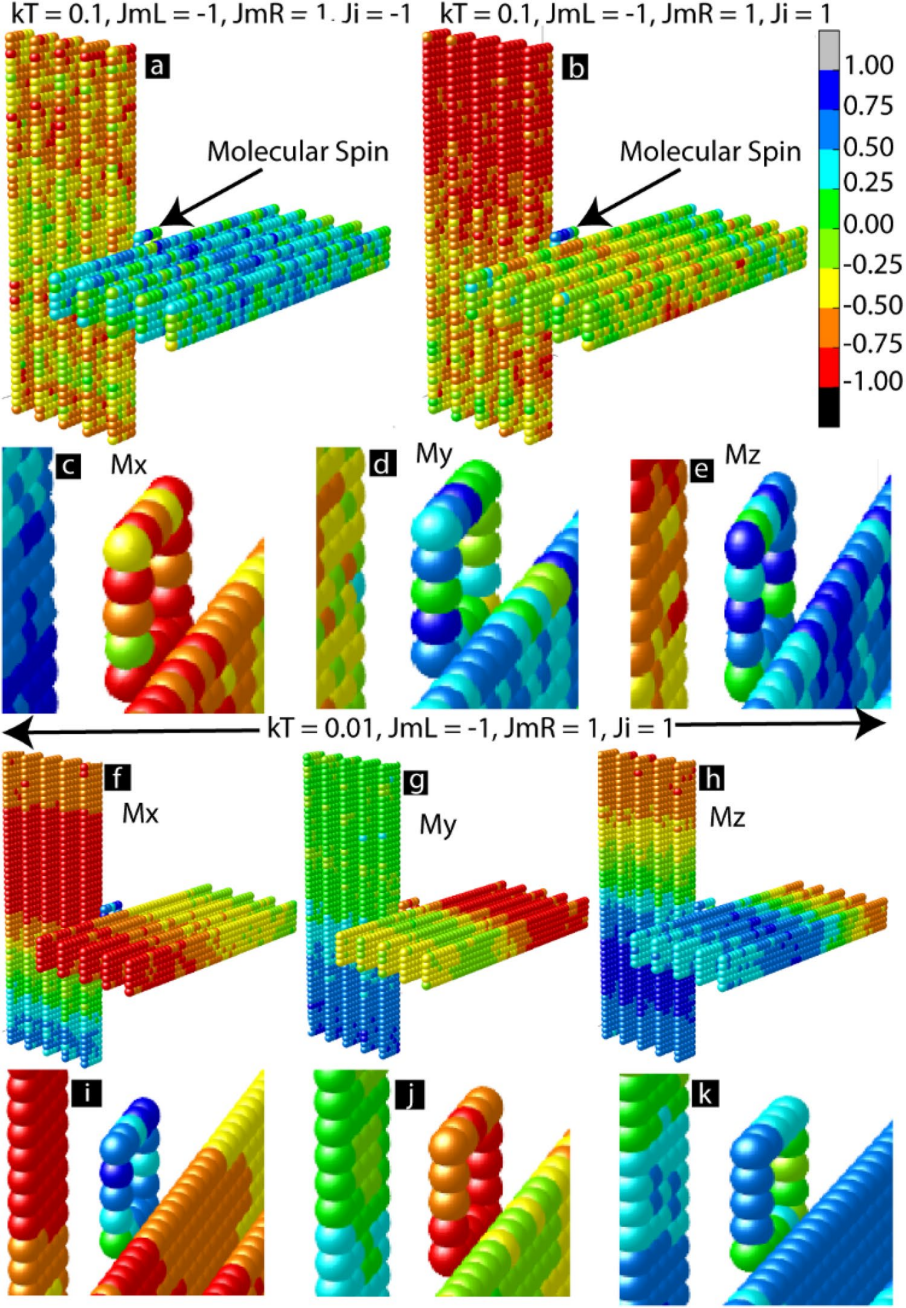
3D Hisenberg model showing equilibrium state with MCS produced color of ferromagnet and molecules. MTJMSD equilibrium state magnetic moment when *Ji* is (**a**) supporting (**b**) opposing the molecule induced antiferromagnetic coupling. (**c**) X, (**d**) Y, and (**e**) Z component molecular magnetic moment as compared to ferromagnet near interface when *Ji* is opposing molecular coupling. At lower temperature (*kT* = 0.01) when *Ji* is opposite to molecular coupling the magnetic moment of MTJMSD in (**f**) X, (**g**) Y, and (**h**) Z direction and magnetic moment near interfaces in (**i**) X, (**j**) Y, and (**k**) Z directions. This figure was plotted in Originlab^®^ 2020 version https://www.originlab.com/ and components were assembled in Adobe Illustrator version available in adobe creative cloud package(https://www.adobe.com/creativecloud.html).

To further investigate the competing *J*_*i*_ and molecule coupling (*J*_*mL*_ and *J*_*mR*_), we investigated the 3D lattice model of MTJMSD (Fig. 5). A 3D lattice for each MTJMSD was captured in the equilibrium state after the simulation duration shown in Fig. 4a. We also investigated if decreasing thermal energy influenced the *J*_*i*_ ability to compete with molecule-induced exchange coupling. At *kT* = 0.1, negative *J*_*i*_ Supported the antiferromagnetic coupling due to the molecule (Fig. 5a). In this case, the two ferromagnetic electrodes aligned antiparallel to each other, and the molecules' magnetic direction matched with one of the ferromagnetic electrodes (Fig. 5a). The closeness of the color corresponding to the magnetic moment of the molecules and first ferromagnetic electrode is because molecules made strong ferromagnetic coupling with the electrode (*J*_*mR*_ = 1). On the other hand, the difference between the color of molecules and the second ferromagnetic electrode is because the molecule made antiferromagnetic coupling with the second electrode (*J*_*mL*_ = − 1).

When *J*_*i*_ opposed the exchange coupling caused by molecules, the magnetic movement of the molecules was random (Fig. 5b). It is noteworthy that MTJMSD typically settled in one of the X, Y, or Z directions after each simulation. In the present case, when defects were opposing the molecular coupling effect, the magnetic movement of the molecules was random in all the X (Fig. 5c), Y (Fig. 5d), and Z (Fig. 5e) directions. It is noteworthy that reducing thermal energy reduced the defect-induced randomness in the magnetic moment of the molecules in X (Fig. 5f), Y(Fig. 5g), and Z (Fig. 5h). At low-temperature, FM electrodes also appeared to attain multiple phases (Fig. 5f–h). We have shown the magnified view of the 3D lattice plots near the interface regions. Due to the appearance of various phases in FM electrodes, we could not conclude a specific direction of MTJMSD's stabilization. Despite less noise in the molecular magnetic moment, molecules' magnetic moment vector alignment was in the X direction (Fig. 5i) and Y direction (Fig. 5k) did not follow the expected alignment that was to be forced by the molecular coupling (i.e., *J*_*mL*_ = − 1, *J*_*mR*_ = 1). Hence, we conclude that even though molecules may be more ordered, they are still impacted by the defect-induced strong exchange coupling.

The impact of molecular spin states in different zones of the MTJMSD under the competing influence of molecular coupling and direct coupling is critical in defining the overall transport attributes. We calculated a correlation factor (*c*) to calculate the range of correlated interaction between the molecule and ferromagnetic state. The correlation factor was calculated by computing the dot product of the spin vector of each atom on an electrode with the average vector of the molecular analogs. The *c* factor was between − 1 to 1. The *c* = 1 represented the strong correlation and the case when molecules and ferromagnet are parallel. Whereas *c* = − 1 indicates that the molecules and ferromagnet are antiparallel.

**Figure 6:**
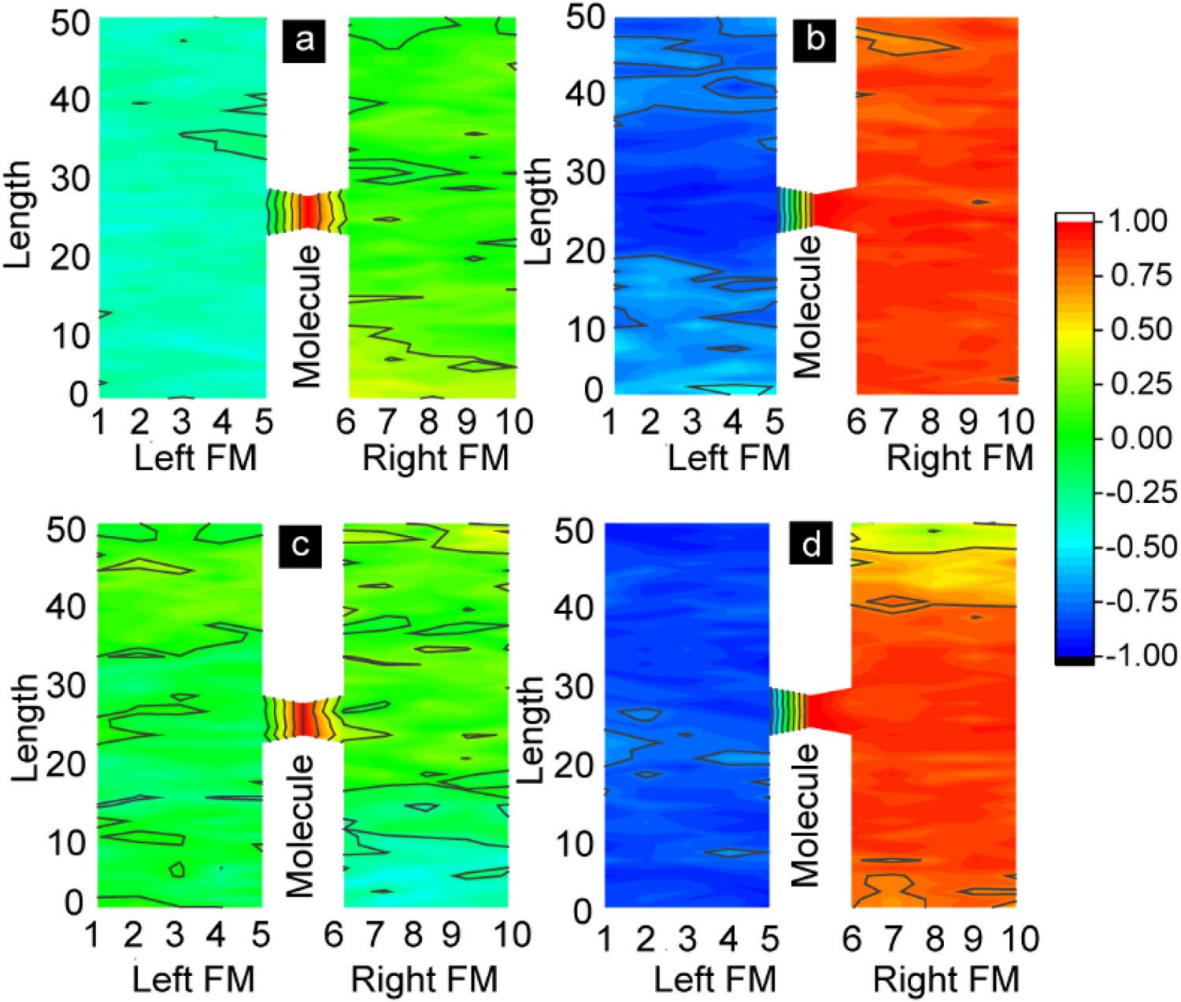
Spatial correlation of molecular spin with ferrmagnets. Vectors along thickness of each ferromagnets are averaged to convert 3D image into 2D image. Correlation factor for MTJMSD with (**a**) *J*_*i*_ = *J*_*mL*_ = *J*_*mR*_ = 0. Correlation factor for *J*_*mL*_ = − 1, and *J*_*mR*_ = 1 molecular cpoupling was plotted for (**b**) *J*_*i*_ = 0, (**c**) *J*_*i*_ = 1, (**d**) *J*_*i*_ = − 1. All data collected at *kT* = 0.1. This figure was plotted in Originlab^®^ 2020 version https://www.originlab.com/ and components were assembled in Adobe Illustrator version available in adobe creative cloud package(https://www.adobe.com/creativecloud.html).

For *J*_*i*_ = *J*_*mL*_ = *J*_*mR*_ = 0, the correlation factor *c* for molecule was fluctuating around zero (Fig. 6a). However, for *J*_*i*_ = 0 and *J*_*mL*_ = − 1, *J*_*mR*_ = 1 molecular spins were highly correlated with left and right FM electrodes (Fig. 6b). In the absence of defect induced coupling, the *J*_*mL*_ = − 1 forced left FM electrode to be antiparallel with respect to molecular spin. The magnitude of *c* was in − 0.75 to − 1 range near the junction area; however, c started decreasing away from the junction (Fig. 6b). However, the right electrode exchibited uniform and highly positive *c* with respect to molecules (Fig. 6b). For *J*_*i*_ = 1 and *J*_*mL*_ = − 1, and *J*_*mR*_ = 1, two electrodes are parallel uncorrelated with the molecular spin (Fig. 6c). It is expected *J*_*i*_ = 1, which is > 20% of the molecular coupling strength, forced two magnetic layers to be parallel with each other. Low *c* magnitude suggest that defect induced molecular coupling produced parallel magnetic state which is ~ 90° with respect to molecular spin state (Fig. 6c). However, antiferromagnetic direct coupling (*J*_*i*_ = − 1) complemented the impact of molecules induced strong antiferromagnetic coupling (*J*_*mL*_ = − 1, *J*_*mR*_ = 1) (Fig. 6d). Right FM electrodes exhibited an intense color region starting from the molecule and ending after the full thickness of the FM electrode (Fig. 6d). In our prior studies, we observed that highly correlated states (*c* = 1) appeared disjointed with the right electrode without the support of direct exchange coupling. The left electrode possessed regions corresponding to *c* in the 0.6 to − 1 range. Interestingly, when *J*_*i*_ was opposite to molecule-induced antiferromagnetic exchange coupling effect, i.e., *J*_*mL*_ = − 1, and *J*_*mR*_ = 1, direct coupling destroyed the molecule correlated states. Uncorrelated molecular and ferromagnetic electrodes are consistent with the 3D lattice images shown in Fig. 5b–e. In this case, molecules were also strongly uncorrelated concerning the two electrodes.

It is noteworthy that many possibilities may arise for a different order of magnitude of direct coupling and molecule-induced exchange coupling. However, due to limited space, a wide-ranging discussion is beyond the scope of this paper. However, we have investigated the change in energy of MTJMSD and interfaces to explore the nature of transitions (Supplementary Fig. S2). The variation in energy was investigated as a function of the molecular coupling strength and direct exchange coupling strength (*J*_*i*_), as discussed in Supplementary Fig. S2.

We also investigated the effect of different ranges of molecular coupling (*J*_*mL*_ and *J*_*mR*_) and direct coupling (*J*_*i*_) to cover a wide range of possibilities. To understand the impact of the varying magnitude of *J*_*i*_, we investigated the equilibrium energy of MTJMSD's Heisenberg model and interfaces as a function of the variation in molecular coupling strengths (*J*_*mL*_ and *J*_*mR*_). The MTJMSD overall energy depends on the combined impact of molecule-induced exchange coupling and direct exchange coupling (Supplementary Fig. S2a).

To demonstrate the direct exchange coupling *J*_*i*_ impact, we have mainly discussed the various molecule-induced ferromagnetic coupling cases. A similar discussion will hold good for molecule-induced antiferromagnetic coupling; the main idea is to show what happened to overall MTJMSD energy when direct coupling changes from the same type (as produced by molecule) to the opposite type. Typically, MTJMSD energy kept increasing as *J*_*i*_ approached near-zero magnitudes. In the absence of molecule-induced exchange coupling (for *J*_*mL*_ = *J*_*mR*_ = 0), MTJMSD's energy was symmetrical around *J*_*i*_ = 0. For stronger molecular coupling after *J*_*mL*_ = *J*_*mR*_ =  ~ 0.2$,$ MTJMSD energy increased at a slower rate for antiferromagnetic type *J*_*i*_, as compared to ferromagnetic type *J*_*i*_ (Supplementary Fig. S2a). Also, with increasing molecular coupling strengths, the apex of MTJMSD's energy curve shifted from 0 to stronger antiferromagnetic *J*_*i*_ (i.e., more negative value of *J*_*i*_). Importantly, the energy reduction over ${J}_{i}$ = 1 to ${J}_{i}$ = − 1 range was very different for different molecular coupling strengths. In the case of *J*_*mL*_ = *J*_*mR*_ = 1, the amount of energy reduction necessary to reach the equilibrium state was ~ 5 units and ~ 40 units for ${J}_{i}$ = − 1 and ${J}_{i}$ = 1, respectively. The energy difference required to reach the equilibrium state provide a plausible reason why antiferromagnetic direct exchange coupling (${J}_{i}$ = − 1) produced highly correlated spatial distribution initiated by the antiferromagnetic molecular coupling (Fig. 5b). Increasing direct coupling strength from 0 to − 1 appears to create entirely new ferromagnetic electrode states compared to strong ferromagnetic molecular coupling. The study of MTJMSD energy suggests a number of important points: (i) if *J*_*i*_ is opposite to molecular coupling nature, then MTJMSD energy will be higher as compared to the case when the nature of *J*_*i*_ and molecular coupling was the same, (ii) *J*_*i*_ also try to reduce the energy of the MTJMSD similar to a molecular coupling, (iii) as a key message we note that *J*_*i*_ ~ − 0.2 can influence ~ five times stronger ferromagnetic molecular exchange coupling effect.

We also investigated direct exchange coupling on the overall MTJMSD's equilibrium energy and interfacial energies. Ferromagnet–ferromagnet interface energy for the extreme cases of molecule induced ferromagnetic (*U*_*i*_ = 1) and antiferromagnetic (*U*_*i*_ = − 1) exchange coupling was determined. For the case of molecule-induced antiferromagnetic coupling (*U*_*i*_ = − 1), the interfacial energy kept increasing from − 1 to 0.2 and then decreased. A similar trend in the form of mirror image was observed for the molecule-induced ferromagnetic coupling (Supplementary Fig. S2b). This study suggests that *J*_*i*_ (direct exchange coupling) has to be around ~ 1/5th of molecular exchange coupling to overcome the molecular exchange coupling impact on MTJMSD. We also studied the effect of *J*_*i*_ on the molecule-ferromagnet interface for the case of molecule-induced antiferromagnetic coupling (Supplementary Fig. S2c). Consistent with the ferromagnet-ferromagnet interface energy profile, ferromagnetic *J*_*i*_ with ~ 1/5th magnitude of molecular exchange coupling produced a transition (Supplementary Fig. S2c). We also varied the strength of molecular coupling to investigate the critical magnitude of *J*_*i*_ necessary to impact MTJMSD. A weaker molecular coupling required decreasing magnitude of the *J*_*i*_ to create a global impact on MTJMSD (Supplementary Fig. S2c).

**Figure 7:**
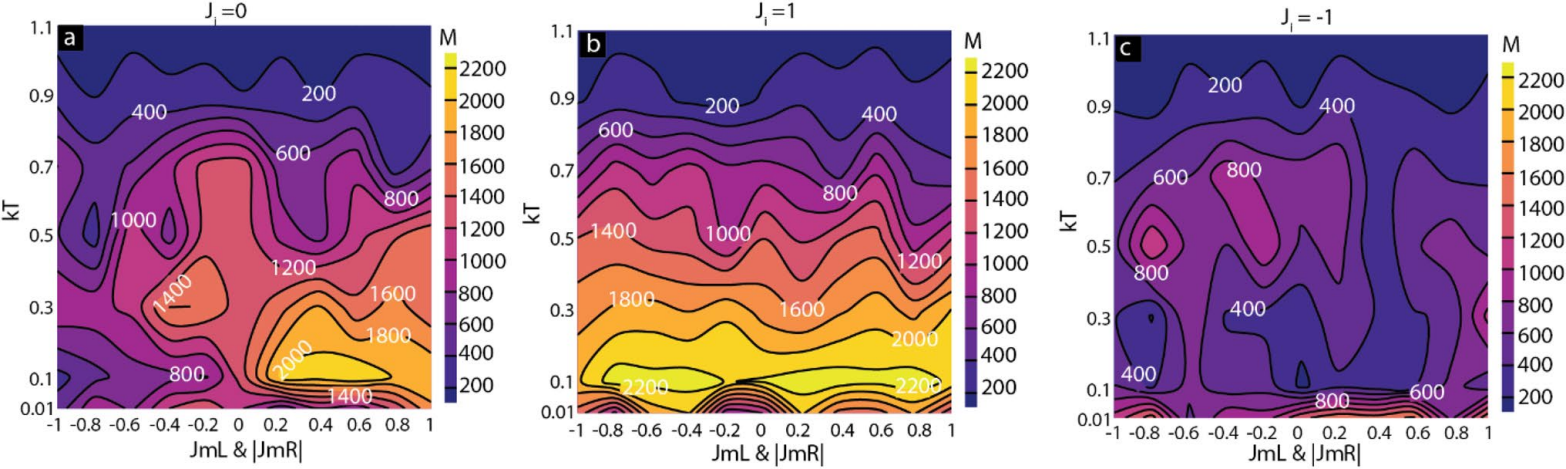
Effect of thermal energy and molecular coupling on MTJMSD magnetic moment under (**a**) no direct Energy, (**b**) ferromagnetic direct coupling, (**c**) antiferromagnetic direct coupling. Simulation data was plotted with the indigenously written Python code https://colab.research.google.com/drive/1ACob9eSsKm3oH4y_X43rjLtVnfockUTv?usp=sharing. All the components were assembled in Adobe Illustrator CS6 version to produce the composite figure.

We also studied thermal energy (*kT*) on the impact of direct coupling and molecular coupling (Fig. 7). For this study, we produced contour plots where color represents the magnetic moment of the MTJMSD under varying *kT* and molecular coupling for the three cases of direct coupling, i.e., *J*_*i*_ = 0, *J*_*i*_ = 1, and *J*_*i*_ = − 1 (Fig. 7a–c). In the absence of direct coupling (*J*_*i*_ = 0), the antiferromagnetic molecular coupling led to a low magnetization state around *kT* = 0.1. Increasing *kT* created thermal disturbance destroying molecular coupling impact and finally leading to a randomized state yielding nearly zero magnetic moments (Fig. 7a). With increasing *kT,* molecule-induced ferromagnetic coupling, i.e., regions of positive *J*_*mL*_ and *J*_*mR*_, the high magnetization state of MTJMSD turned into a randomized spin state. As a result, MTJMSD settled into a zero magnetic moment (Fig. 7a). For *kT* < 0.1, defect-free MTJMSD also settled in the relative low magnetizattion state (1400 vs. 2000) for the ferromagnetic coupling regime (Fig. 7a). The molecule-induced coupling impact was largely defeated for strong direct ferromagnetic exchange coupling (*J*_*i*_ = 1) (Fig. 7b). The MTJMSD generally stayed in the high magnetic moment state for 0.1 to 0.3 *kT* range (Fig. 7b). Similarly, for the case of strong direct antiferromagnetic coupling (*J*_*i*_ = − 1), MTJMSD's magnetic moment was generally low for all the cases of molecular coupling strength at low temperature (Fig. 7c). The lowest case of MTJMSD magnetic moment was observed for *J*_*mL*_ = − 1 and *J*_*mR*_ = 1 near *kT* = 0.1 (Fig. 7c) and consistent with the corresponding 3D lattice of MTJMSD shown in Fig. 7b. With increasing *kT*, MTJMSD transcended into a low magnetization state. Interestingly, a relatively low magnetization regime was observed for *kT* < 0.1 and *J*_*i*_ = 1 around the 0.8 antiferromagnetic coupling (i.e., *J*_*mL*_ and |*J*_*mR*_| = − 0.8) (Fig. 7b). A similar low magnetic moment regime was observed for − 0.4 < *J*_*mL*_ and |*J*_*mR*_|< 0.2 range for low thermal energy (Fig. 7b). Such regimes were absent for low thermal energy for *J*_*i*_ = 0 (Fig. 7a) and *J*_*i*_ = − 1 (Fig. 7c). We surmise that this state is due to the holistic homeostasis resulting from the combination of multiple variables. This resaerch suggests that molecule impact will vary dramatically with temperature.

**Figure 8:**
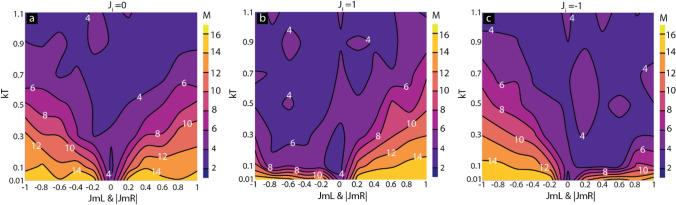
Effect of thermal energy and molecular coupling on molecular magnetic moment under (**a**) no direct Energy, (**b**) ferromagnetic direct coupling, (**c**) antiferromagnetic direct coupling. Simulation data was plotted with the indigenously written Python code https://colab.research.google.com/drive/1ACob9eSsKm3oH4y_X43rjLtVnfockUTv?usp=sharing. All the components were assembled in Adobe Illustrator CS6 version to produce the composite figure.

We also investigated the impact of direct coupling and molecular coupling on the molecular magnetic moment at different thermal energies (Fig. 8). For *J*_*i*_ = 0, the molecular magnetic moment was symmetrical around zero molecular coupling strength (Fig. 8a). This data is justifiable because direct exchange coupling is unavailable. However, for *J*_*i*_ = 1, the molecular magnetic moment remains in a higher state (Fig. 8b). Interestingly, the strong molecule-induced antiferromagnetic coupling promoted the higher magnetic moment near low thermal energy despite strong *J*_*i*_ (Fig. 8b). A similar trend was observed for the strong and direct ferromagnetic coupling (Fig. 8c). It appears that at low *kT,* molecules are able to assume an ordered state despite strong *J*_*i.*_. For *J*_*i*_ = 1 and *kT* < 0.1 cases, molecules are highly ordered for *J*_*mL*_ and |*J*_*mR*_|> 0.2 and more ordered than the case of *J*_*mL*_ and |*J*_*mR*_| < − 0.2 (Fig. 8b). For *J*_*i*_ = 1 and *kT* < 0.1 cases, MTJMSD's low magnetic moment (Fig. 7b) is caused by low temperature stable molecular ordered state (Fig. 8b).

The theoretical discussion in this paper is relevant to our experimental observations and will impact the future application of MTJMSD showing high TMR. Experimentally we observed that applied current change on a stable MTJMSD, where molecule induced strong exchange coupling, generally impacted the current state and promoted high current state^8,14^. It was also observed that an MTJMSD was much more robust than the MTJ testbed against degradation due to current flow. However, in the initial state, MTJMSD exhibited significant resistance change due to current and generally promoted a temporary high current state^14^. We hypothesize that molecular channels provide a dominant medium for charge transport and reduce the transport via a stable tunneling barrier. However, on an unstable MTJMSD, current flow accelerated the degradation of the MTJMSD and finally produced a permanent high current state. We believe that if a bare MTJ possessed a large population of defects, then molecular channels could not divert the electron flow and a major transport occurred via defects. The current flow via defects resembles a high positive *J*_*i*_ with > 20% of the molecular exchange coupling (*J*_*mL*_ and *J*_*mR*_). Transport via defects expected to create hot spots for very high transport. Transport via defects also modifies the nature of defects^26,36^ and increases defect populations resulting in a failed MTJMSD. Future MTJMSD focusing on spin-transfer torque mechanism, where transport via MTJ is necessary, must ensure that coupling via defects is minimized to produce stable devices.

## Conclusions

This paper focused on the molecule-induced exchange coupling in the presence of direct coupling via space between the two ferromagnetic electrodes. We reported experimental observations of stress-induced pinhole and hillocks type defects in the alumina (AlOx) tunnel barrier. Other tunnel barriers like MgO may have point defects. As a significant conclusion, every tunnel barrier for MTJMSD is expected to have some form of imperfection. We have demonstrated FMR as a tool to identify the presence of defects. Defects within tunnel barriers may lead to an inter-electrode exchange coupling competing with the molecular channels. Direct coupling between ferromagnetic electrodes either supported or canceled the impact of molecule-induced exchange coupling. We performed an MCS study to understand the impact of defect-induced exchange coupling on molecule-induced exchange coupling. The effect of defects was dependent on the thermal energy and molecular coupling. As thermal energy decreased, the impact of defects decreased, and molecules could still show the effect. Molecules in MTJMSD could still maintain correlated states at lower thermal energy. The mechanism behind the impact of direct exchange coupling was based on destroying the coherent molecular spin orientation in MTJMSD. We observed that *J*_*i*_ =  ~ 0.2 sufficient to disturb the five times stronger molecule induced strong antiferromagnetic coupling suggesting that the low magnitude of *J*_*i*_ can defeat the strong molecule coupling effect. This paper also indicates that an MTJMSD does not have to have the perfect insulator to produce molecular devices. MTJMSD with *J*_*i*_ $< 0.2$ are expected to have somewhat dormant defects. We also provided evidence that even with high *J*_*i*_ strength, molecular nanostructures can maintain an ordered state for low thermal energy. Future studies will focus on investigating the effect of *J*_*i*_ in the presence of other magnetic interactions and magnetic electrode properties. In future work, we also plan to focus on tunneling magnetoresistance (TMR) properties of MTJMSD based on our experimental and theoretical understanding of the role of defects.

## Supplementary Information

Supplementary Information.

## Data Availability

The data that support the findings of this study are available from the corresponding author upon reasonable request.
