# Supplementary material for: Molecular coupling competing with defects within insulator of the magnetic tunnel junction-based molecular spintronics devices
Source: Sci Rep. 2021 Aug 24;11:17128. doi: 10.1038/s41598-021-96477-3 (PMC8384883; doi:10.1038/s41598-021-96477-3)

Supplementary Material

**Molecular Coupling Competing with Defects Within Insulator of the Magnetic Tunnel Junction based Molecular Spintronics Devices**

*Pawan Tyagi*, Hayden Brown, Andrew Grizzle, Christopher D’Angelo, Bishnu R. Dahal,*

Center for Nanotechnology Research and Education, Mechanical Engineering, University of the District of Columbia, Washington DC-20008, USA

Corresponding Author Email: ptyagi@udc.edu

Fig. S1: Current-voltage (I-V) response from a MTJ with defective tunnel barrier in (a) bare state and (b) after interaction with molecules. (c) I-V response from a stable MTJ before and after becoming MTJMSD. (d) A MTJMSD based on good MTJ attained stable suppressed equilibrium current state.


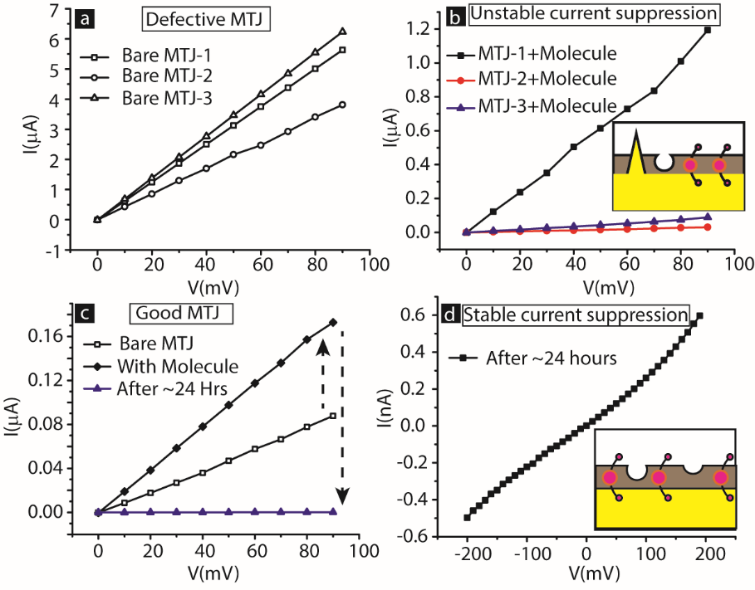


**FIG. S2.** Energy as a function of the molecular coupling strengths $JmL/JmR$ and $J_{i}$ for (a) whole MTJMSD device, (b) direct ferromagnetic interfaces due to $J_{i}$ and (b) two molecule-ferromagnet interfaced due to $JmL$ and $JmR$ .


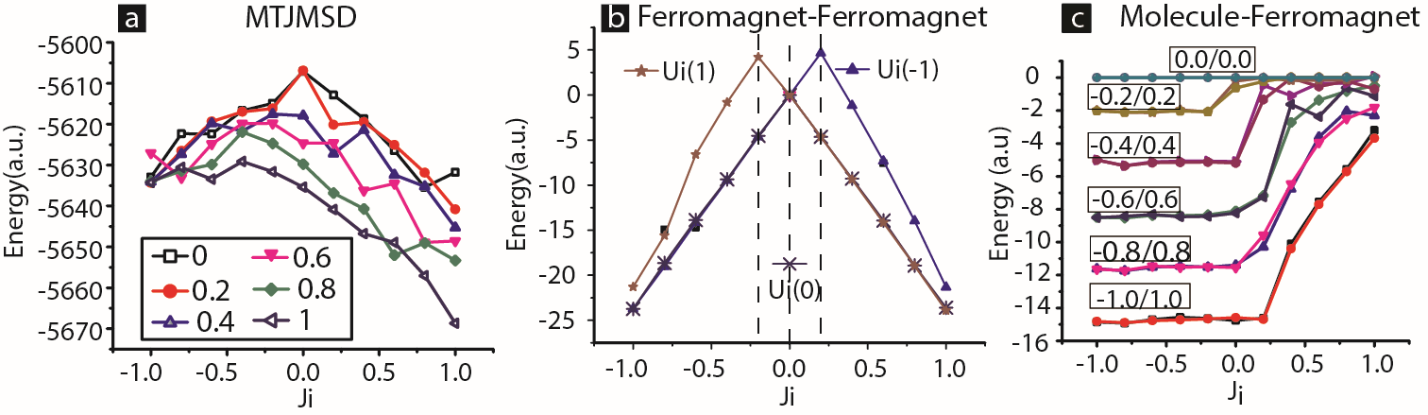

Supplement: Supplementary file 1 — Supplementary Information. [file 41598_2021_96477_MOESM1_ESM.docx]
